# Serum levels of miR-29, miR-122, miR-155 and miR-192 are elevated in patients with cholangiocarcinoma

**DOI:** 10.1371/journal.pone.0210944

**Published:** 2019-01-17

**Authors:** Sven H. Loosen, Georg Lurje, Georg Wiltberger, Mihael Vucur, Alexander Koch, Jakob N. Kather, Pia Paffenholz, Frank Tacke, Florian T. Ulmer, Christian Trautwein, Tom Luedde, Ulf P. Neumann, Christoph Roderburg

**Affiliations:** 1 Department of Medicine III, University Hospital RWTH Aachen, Aachen, Germany; 2 Department of Visceral and Transplantation Surgery, University Hospital RWTH Aachen, Aachen Germany; 3 Division of Gastroenterology, Hepatology and Hepatobiliary Oncology, University Hospital RWTH Aachen, Aachen, Germany; 4 Department of Urology, University Hospital Cologne, Cologne, Germany; 5 Department of Surgery, Maastricht University Medical Center (MUMC), Maastricht, The Netherlands; Karolinska Institutet, SWEDEN

## Abstract

**Objectives:**

Cholangiocarcinoma (CCA) represents the second most common primary hepatic malignancy. Despite tremendous research activities, the prognosis for the majority of patients is still poor. Only in case of early diagnosis, liver resection might potentially lead to long-term survival. However, it is still unclear which patients benefit most from extensive liver surgery, highlighting the need for new diagnostic and prognostic stratification strategies.

**Methods:**

Serum concentrations of a 4 miRNA panel (miR-122, miR-192, miR-29b and miR-155) were analyzed using semi-quantitative reverse-transcriptase PCR in serum samples from 94 patients with cholangiocarcinoma undergoing tumour resection and 40 healthy controls. Results were correlated with clinical data.

**Results:**

Serum concentrations of miR-122, miR-192, miR-29b and miR-155 were significantly elevated in patients with CCA compared to healthy controls or patients with primary sclerosing cholangitis without malignant transformation. Although preoperative levels of these miRNAs were unsuitable as a prognostic marker of survival, a strong postoperative decline of miR-122 serum levels was significantly associated with a favorable patients’ prognosis.

**Conclusions:**

Analysis of circulating miRNAs represents a promising tool for the diagnosis of even early stage CCA. A postoperative decline in miRNA serum concentrations might be indicative for a favorable patients’ outcome and helpful to identify patients with a good prognosis after extended liver surgery.

## Introduction

Compared to other gastrointestinal cancers, cholangiocarcinoma (CCA) is a comparatively rare malignancy originating from the bile ducts [1]. Besides a relatively high prevalence in some parts of Asia, CCA shows an incidence rate of 2-3/100,000 people in the United states and most parts of Europe [2]. However, the global incidence rates are increasing over the last decades [3], leading to a progressive awareness of this disease in the field of gastrointestinal oncology. Apart from liver transplantation, surgical tumor resection has remained the only potentially curative treatment option in daily practice but only few patients qualify for surgery due to advanced stage of disease at diagnosis [4]. Thus, early detection of CCA is essential to provide patients with the best possible therapeutic approach in order to improve the patients’ prognosis. However, the scientific effort of the last years have failed to establish appropriate biomarkers for this purpose and the clinically most widely used tumor markers such as CEA and CA19-9 have only a limited diagnostic power [5,6]. Therefore, novel classes of biomarkers are urgently needed in the diagnostics and treatment of patients with CCA.

MiRNAs represent a large group of endogenous, non-coding RNA molecules with a length of ~22 nucleotides [7,8]. They are created by a complex multi-step process in the cell nucleus and cytoplasm and have been associated with various cell regulation processes such as cell metabolism, cell growth and differentiation as well as cell death [9–11]. Due to their simple chemical structure and the resulting biological stability, circulating miRNAs have been suggested as potential biomarkers for several diseases including inflammatory diseases [12], acute liver failure [13] and cancer [14,15]. As such, different circulating miRNAs have been suggested as diagnostic or prognostic biomarkers for gastrointestinal malignancies such as pancreatic adenocarcinoma and hepatocellular carcinoma [16–19].

In cholangiocarcinoma, a series of miRNAs including miR-21 and miR-221 were found to be overexpressed in tumor samples [20]. However, only a few studies on the diagnostic and prognostic role of circulating miRNAs exist and the scientific validity is often restricted due to small CCA cohort sizes [20,21]. Therefore, we aim to evaluate the diagnostic and prognostic power of a panel of four circulating miRNAs (miR-122, miR-192, miR-29b, and miR-155) in a large and well-characterized cohort of CCA patients undergoing tumor resection in our university hospital.

## Materials & methods

### Study design and patient characteristics

This observational cohort study was designed to evaluate circulating miRNAs as a diagnostic and prognostic serum marker in patients undergoing resection of cholangiocarcinoma. Of all patients admitted to the Department of visceral and transplantation surgery at the University Hospital RWTH Aachen for surgical resection of CCA between 2011 and 2015, a subgroup of n = 94 patients who agreed to participate in our study were prospectively recruited (see Table 1 for details). Serum samples were collected prior to surgery and 6–7 days after tumor resection. As a control population we analyzed a total of 40 healthy, cancer-free blood donors with normal values for blood counts, C-reactive protein and liver enzymes. These patients were medically examined on any sign of disease on a regular basis. Moreover, 10 patients with primary sclerosing cholangitis (PSC) and no sign of malignant transformation were enrolled into the study. PSC patient were enrolled in a surveillance protocol for malignant transformation including routine laboratory test (tumor markers and liver parameters) and abdominal ultrasound or CT/MRI scans when indicated. The study protocol was approved by the local ethics committee and conducted in accordance with the ethical standards laid down in the Declaration of Helsinki (ethics committee of the University Hospital Aachen, RWTH University, Aachen, Germany). Written informed consent was obtained from the patient.

**Table 1:** Characteristics of study population.

	CCA patients	Healthy controls	PSC patients
Number of patients/controls	**94**	**40**	**10**
Gender [%]:			
*male-female*	**57–43**	**76.3–23.7**	**80–20**
Age [years, median and range]	**67 [37–84]**	**37.5 [19–74]**	**35 [23–49]**
BMI [kg/m2, median and range]	**25.51 [19.15–46.36]**	**-**	**-**
Anatomic location of CCA [%]			
*Intrahepatic*	**37.2**	**-**	**-**
*Klatskin*	**42.6**	**-**	**-**
*Distal*	**11.7**	**-**	**-**
*Gallbladder*	**8.5**	**-**	**-**
Staging and grading [%]			
*T1-T2-T3-T4*	**8.5–36.6–31.7–23.2**	**-**	**-**
*N0-N1*	**43.8–56.3**	**-**	**-**
*M0-M1*	**79.5–20.5**	**-**	**-**
*G2-G3*	**66.4–33.6**	**-**	**-**
*R0-R1*	**58.2–41.8**	**-**	**-**
Portal vein embolization [%]			
*Yes-No*	**16.9–83.4**	**-**	**-**
ECOG Performance status [%]			
*0-1-2-3-4*	**53.4–37.0–9.6-0-0**	**-**	**-**
Deceased during follow-up [%]			
*Yes-No*	**34.0–66.0**	**-**	**-**

### MiRNA isolation from Serum

300 μl serum was spiked with *miScript miRNA mimic SV40* (Qiagen, Germany) for sample normalization. 600 μl *peqGOLD TriFast* (VWR) and 150 μl chloroform were added to the sample and mixed vigorously for 15 sec followed by an incubation at room temperature for 10 min. Samples were centrifuged for 15 min at 12,000 g until complete phase separation. The aqueous phase, containing total RNA, was precipitated with 375 μl 100% isopropanol and 1.5 μl glycogen (Fermentas, St. Leonroth, Germany) overnight at -20°C. After centrifugation at 4°C for 30 min (12,000 g) the pellets were washed once with 70% ethanol and centrifugation at 12000 g, 5 min and 4°C. Precipitated RNA was resuspended in 30 μl RNase free water.

### Semi-quantitative reverse transcriptase PCR

Total RNA was used to synthesize cDNA utilizing miScript Reverse Transcriptase Kit (Qiagen) according to the manufacturer’s protocol, and was resuspended in suitable amounts of H_2_O. cDNA samples (2 μl) were used for semi-quantitative PCR in a total volume of 25 μl using the miScript SYBR Green PCR Kit (Qiagen) and miRNA specific primers (Qiagen) on a PCR machine (Applied Biosystems 7300 Sequence Detection System, Applied Biosystems, Foster City, CA). Data using the 2^-ΔΔCT^ method were presented as relative gene expression. Data were generated and analyzed using the SDS 2.3 and RQ manager 1.2 software packages. Data were generated and analyzed using the SDS 2.3 and RQ manager 1.2 software packages (Applied Biosystems).

### Statistical analysis

Statistical analyses have been performed as recently described in detail [22]. In brief, serum data are given as median and range to reflect the skewed distribution of analysis on human samples. Shapiro-Wilk-Test was used to test for normal distribution. Non-parametric data were compared using the Mann-Whitney-U-Test for two independent variables and the Kruskal-Wallis-H-Test for multiple independent variables. For related samples (e.g. pre- and post-OP) the Wilcoxon signed-rank test was used. Box plot graphics display a statistical summary of the median, quartiles and ranges. ROC curves were generated by plotting sensitivity against 1-specificity. The optimal cut-off values for ROC curves were defined by the Youden-Index (YI = sensitivity + specificity—1). Statistical differences between ROC curves were analyses by the *DeLong* method [23]. Kaplan-Meier curves were plotted to display the impact on survival. Log-rank test was used to test for differences between subgroups in Kaplan-Meier curve analysis. All statistical analyses were performed with SPSS 23 (SPSS, Chicago, IL, USA) [24]. A p-value of < 0.05 was considered statistically significant (* p < 0.05; ** p < 0.01; *** p < 0.001).

## Results

### Serum levels of miR-122, miR-192, miR-29b, and miR-155 are elevated in patients with cholangiocarcinoma

**Fig 1:**
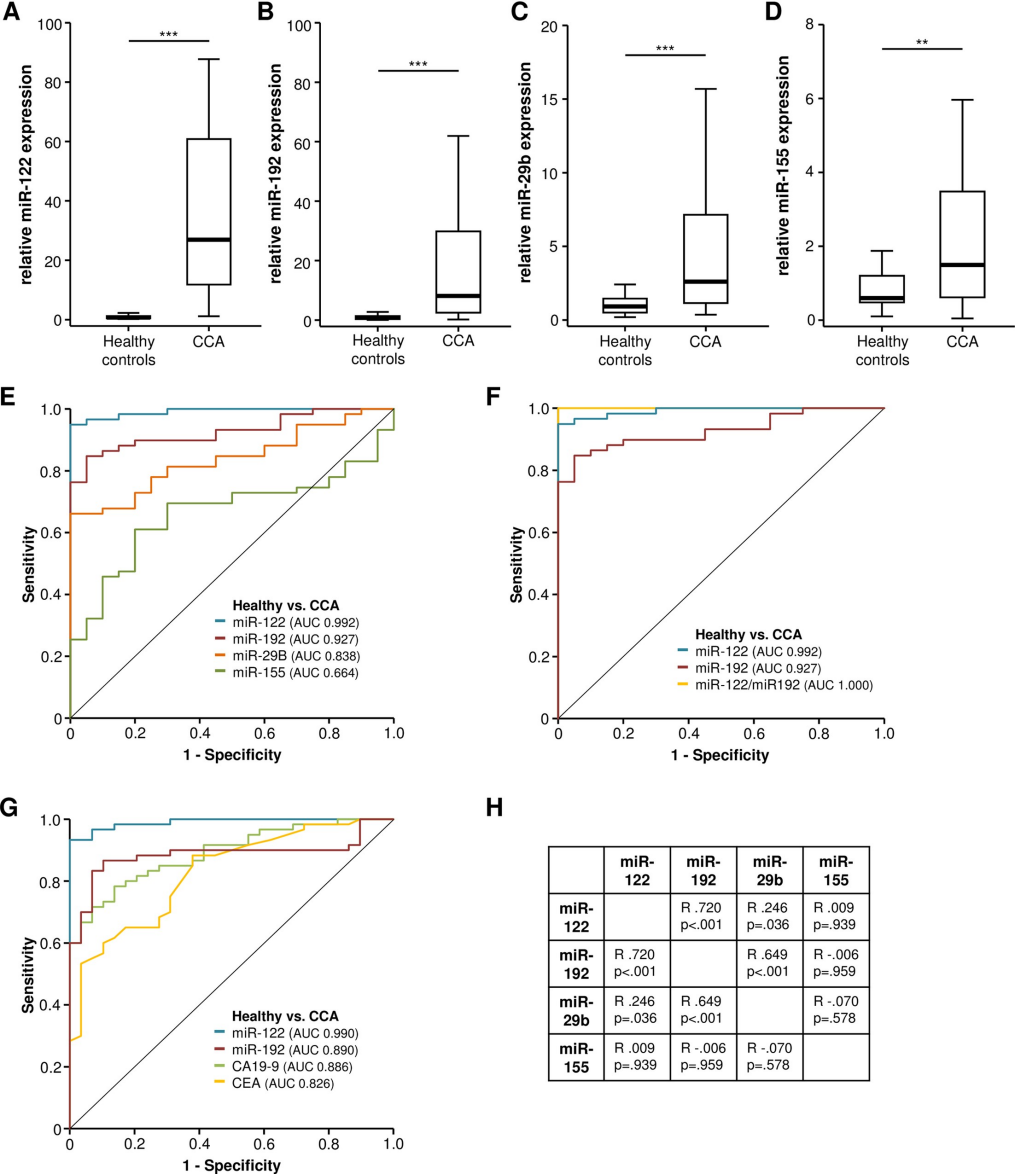
Serum levels of miR-122, miR-192, miR-29b, and miR-155 are elevated in patients with cholangiocarcinoma. Circulating levels of miR-122 (A), miR-192 (B), miR-29b (C) and miR-155 (D) are significantly elevated in CCA patients compared to healthy controls (U-Test). ROC curve analyses show a superior diagnostic potential for miR-122 and miR-192 regarding the discrimination between CCA patient and healthy controls (E, *DeLong* test). The combination of two miRNAs (miR-122 and miR-192) has a superior diagnostic potential compared to either miRNA alone (F). MiR-122 has a significantly higher AUC value compared to CA19-9 (p = 0.006) and CEA (p<0.001, G, *DeLong* test). Serum levels of miR-122, miR-192 and miR-29b show a significant positive correlation with each other (H).

To evaluate a potential role of serum miRNAs as a diagnostic biomarker for CCA, we first compared circulating levels of the different miRNAs in CCA patients with those in healthy controls (patients characteristics are given in Table 1). In this analysis, circulating levels of miR-122 and miR-192 were significantly elevated (median expression > 5-fold) in CCA patients compared to controls (Fig 1A and 1B; Table 2). Similarly, but to a lesser extend (median expression < 5-fold), serum levels of miR-29b and miR-155 were also significantly elevated in patient with CCA (Fig 1C and 1D; Table 2). We next performed ROC curve analysis to evaluate the diagnostic power of the investigated miRNAs to distinguish between CCA patients and healthy controls. Here, we found a superior diagnostic potential for miR-122 and miR-192, which showed an AUC of 0.992 and 0.927, while miR-29b and miR-155 displayed inferior AUC values of 0.838 and 0.664 (Fig 1E). Statistical comparison between the ROC curves by the *DeLong* method [23] revealed a significantly higher AUC value for miR-122 (p = 0.001) and miR-192 (p = 0.014) compared to miR-29b as well as miR-155. We next compared the diagnostic sensitivity of each miRNA at a defined specificity of 90%. In line with the previous findings, miR-122 showed the highest sensitivity of 96.6%, followed by miR-192 (86.4%), miR-29b (67.8%) and miR-155 (45.8%). We next established ideal cut-off values using the Youden-Index method to calculate the optimal diagnostic sensitivity and specificity. At these optimal diagnostic cut-off values, the maximum sensitivity/specificity for miR-122 and miR-192 was 94.9/100% and 84.7/95.0%, respectively. For miR-29b and miR-155 a maximum sensitivity/specificity of 66.1/100% and 61.0/80.0% was found. Hypothesizing that the combinational use of more than one miRNA might even further increase the diagnostic power, we subsequently stratified patients into 5 groups according to their seropositivity (above the ideal cut-off value) for one or more miRNA (0: seronegative for all four miRNAs to 4: seropositive for all four miRNAs). Importantly, this combined analysis revealed a superior diagnostic power showing a sensitivity of 98.3% and a specificity of 100% for the discrimination between CCA patients and healthy controls when at least 2 miRNAs were seropositive. In line, ROC curve analysis revealed that the combination of more than one miRNA by binary logistic regression analysis (e.g. miR-122 and miR-192) had a superior diagnostic power, than either miRNA alone (Fig 1F). Multivariate binary logistic regression analysis further showed that the diagnostic relevance of miR-122 and miR-192 was independent of the patients liver function (AST) or cholestasis (ALP, S1 Table).

**Table 2:** Serum levels of the investigated miRNAs and other clinically relevant parameters.

	CCA patients median (range)	PSC patients median (range)	Healthy controls median (range)
miR-122 pre-OP	26.91 (1.14–4824.03)	4.96 (0.98–34.67)	0.69 (0.20–4.27)
miR-192 pre-OP	8.11 (0.17–9324.65)	1.84 (0.30–11.30)	0.82 (0.14–5.46)
miR-29b pre-OP	2.60 (0.35–734.19)	0.838 (0.43–1.97)	0.91 (0.18–2.41)
miR-155 pre-OP	1.49 (0.452–1542.38)	0.32 (0.20–0.64)	0.60 (0.10–3.44)
miR-122 post-OP	13.86 (0.02–107.39)	-	-
miR-192 post-OP	4.90 (0.82–317.02)	-	-
miR-29b post-OP	2.36 (0.38–36.49)	-	-
miR-155 post-OP	3.05 (0.65–184.07)	-	-
CEA pre-OP [ng/ml] ULN: 5.0 ng/ml	3.35 (0.71–333.0)	1.60 (0.20–3.90)	1.40 (0.30–6.30)
CA19-9 pre-OP [U/ml] ULN: 34.0 U/ml	90.0 (0.60–22911.0)	14.1 (4.0–78.4)	6.30 (0–44.1)
AST pre-OP [U/l] ULN: 50 U/l	47.0 (17.0–1587.0)	-	28.0 (20.0–78.0)
ALT pre-OP [U/l] ULN: 50 U/l	44.0 (10.0–1097.0)	-	20.0 (5.0–82.0)
Bilirubin pre-OP [mg/dl] ULN: 1.2 mg/dl	1.10 (0.24–21.49)	-	0.44 (0.10–1.46)
GGT pre-OP [U/l] ULN: 60.0 U/l	297.5 (36.0–2015.0)	-	18.0 (8.0–120.0)
ALP pre-OP [U/l] ULN: 130.0 U/l	217.0 (52.0–1055.0)	-	70.0 (40.0–102.0)
CRP pre-OP [mg/l] ULN: 5.0 mg/l	15.3 (0.0–230.0)	-	-
Creatinine pre-OP [mg/dl] ULN: 1.2 mg/dl	0.8 (0.42–1.62)	-	-

ALT: Alanine-Aminotransferase, ALP: Alkaline phosphatase, AST: Aspartat-Aminotransferase, CA19-9: Carbohydrate antigen 19–9, CEA: Carcinoembryonic antigen, CRP: C-reactive protein, GGT: Gamma-glutamyltransferase, miR: microRNA, ULN: upper limit of normal

Subsequently, we compared the diagnostic power of miR-122 and miR-192 with clinically established tumor markers such as CEA and CA19-9 [25,26]. Importantly, this analyses revealed that circulating levels of miR-122 had a significantly higher AUC value compared to both CA19-9 (p = 0.006) and CEA (p<0.001), while circulating levels of miR-192 had a similar diagnostic power compared to the established tumor markers (Fig 1G).

Moreover, we performed correlation analysis to unravel potential co-regulations between the investigated miRNAs. Interestingly, we observed a strong positive correlation between miR-122, miR-192 and miR29b in CCA patients, while circulating levels miR-155 did not correlate with any of the other analyzed miRNA (Fig 1H). Importantly, circulating levels of all four miRNAs as well as CA19-9 did not correlate with markers of cholestasis (bilirubin, GGT, ALP).

### Circulating levels of miRNAs can discriminate between benign and malignant hepatobiliary disease

**Fig 2:**
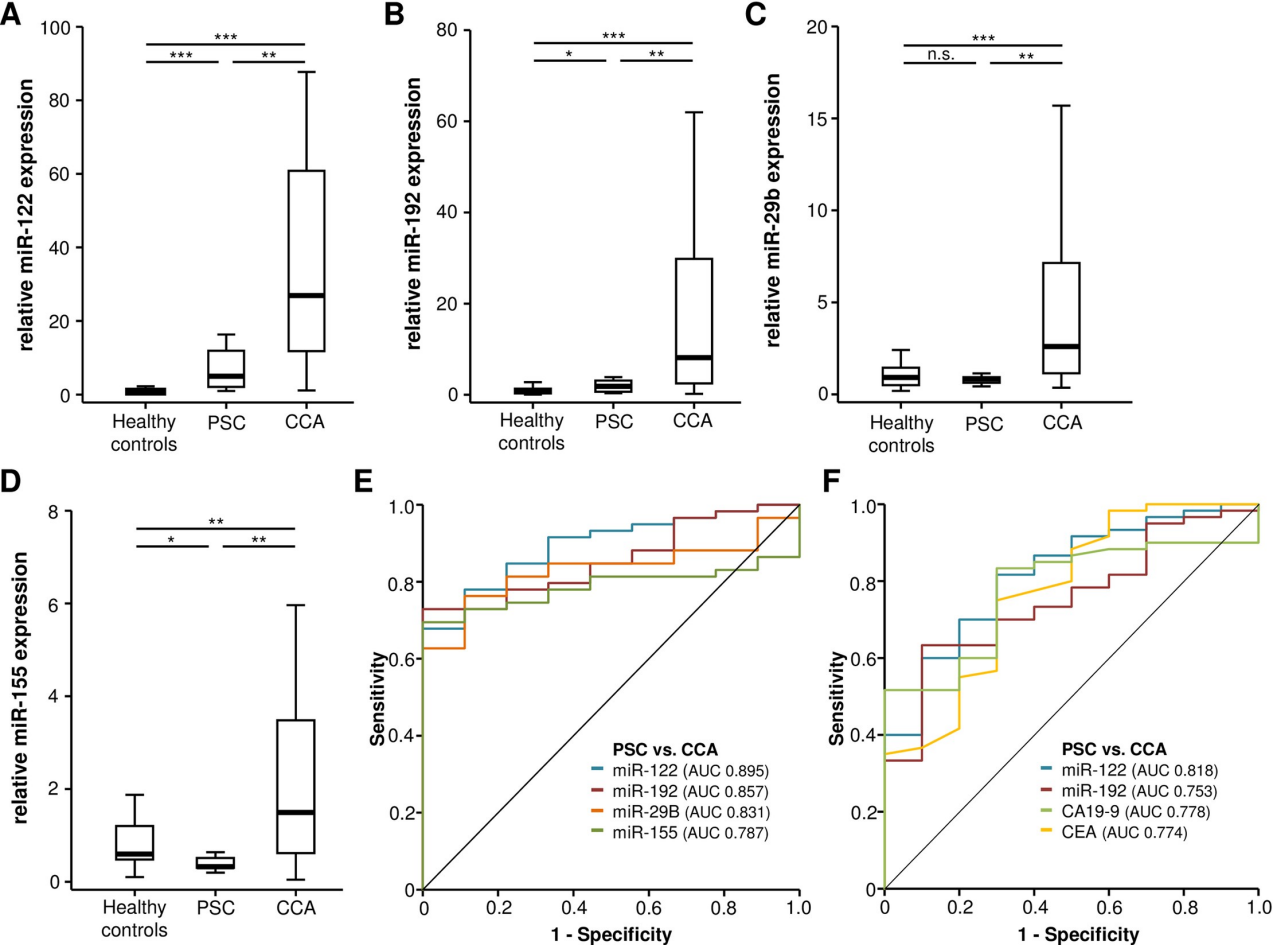
Circulating levels of miRNAs can discriminate between benign and malignant hepatobiliary disease. Serum levels of miR-122 (A) and miR-192 (B) are significantly elevated in PSC patients compared to healthy controls but significantly lower compared to CCA patients (U-Test). PSC patients show similar levels of miR-29b (C) and decreased levels of miR-155 compared to healthy controls (D, U-Test). ROC curve analysis reveals that miR-122 has the highest AUC value for the discrimination between PSC and CCA patients (E). miR-122 and miR-192 show comparable AUC values when compared to standard tumor markers of CCA such as CEA and CA19-9 (F, *DeLong* test).

As circulating levels of various miRNAs have been associated with a number of non-malignant hepatobiliary diseases such as viral hepatitis, NASH, primary sclerosing cholangitis or primary biliary cirrhosis [27–30], we next analyzed serum levels of miR-122, miR-192, miR-29b and miR-155 in a small cohort of patients with primary sclerosing cholangitis (PSC) that did not show any sign of malignant transformation (n = 10). In line with previous findings, serum levels of miR-122 were significantly elevated in PSC patients compared to healthy controls (Fig 2A). However, the relative miR-122 expression in these PSC patients was still significantly lower compared to CCA patients (5-fold vs. 27-fold median miR-122 expression, Fig 2A), arguing that miR-122 might still be a potential biomarker for the diagnosis of CCA even in the context of benign cholangiopathies. Similar results were obtained for circulating levels of miR-192, which showed a slight elevation in PSC patients compared to healthy controls but significantly lower serum levels compared to CCA patients (Fig 2B). Moreover PSC patients showed similar levels of miR-29b and even decreased levels of miR-155 compared to healthy controls (Fig 2C and 2D), suggesting that a rise in serum miR-155 levels might reflect a specific process that only occurs during malignant transformation but not during inflammatory reaction of the biliary system. However, as the up-regulation of miR-155 in our cohort of patients was distinctly lower compared to miR-122 and miR-192, ROC curve analysis revealed that miR-122 still has the best diagnostic power for the discrimination between PSC and CCA patients (AUC_miR-122_ 0.895, AUC_miR-192_ 0.857, AUC_miR-29b_ 0.831 and AUC_miR-155_ 0.787, Fig 2E). Finally, when comparing the diagnostic potential for the discrimination between CCA and PSC of the different miRNAs with established CCA tumor markers, ROC curve analysis showed that miR-122 (AUC: 0.818) had a slightly higher AUC value compared to CA19-9 (AUC: 0.778) and CEA (AUC: 0.774), while miR-192 had a comparable AUC value (0.753) (Fig 2F). However, statistical comparison of AUC values by *DeLong* method did not reveal a significant difference between all four markers.

### MiRNA serum levels do not reflect disease characteristics

Subsequently, we evaluated if serum levels of the investigated miRNAs might reflect different disease characteristics such as tumor localization, the TNM stage or the tumor grading. However, no significant differences in serum miRNA levels became apparent between patients with intrahepatic CCA, Klatskin tumor, distal CCA or gallbladder carcinoma (S1A–S1D Fig). Similarly, levels of circulating miR-122, miR-192, miR-29b and miR-155 were unaltered between patients with different T-status (T1-4, S1E–S1H Fig), nodal negative and positive (N0 vs. N1, S1I–S1L Fig) as well as non-metastasized and metastasized disease (M0 vs. M1, S1M–S1P Fig). The tumor grading (moderately differentiated, G2 vs. poorly differentiated, G3) had also no influence on the levels of serum miRNAs (miR-122: p = 0.637, miR-192: p = 0.276, miR-29b: 0.703, miR-155: p = 0.1000, U-test). Finally, we compared preoperative miRNA levels in patients with complete R_0_ resection and R_1_-resected patients. However, no significant difference became apparent between these groups (miR-122: p = 0.204, miR-192: p = 0.105, miR-29b: 0.310, miR-155: p = 0.414, U-test).

In order to exclude a potential bias of liver inflammation and/fibrosis on circulating miRNA levels, we compared miRNA serum levels between patients who presented with histological signs of liver inflammation or fibrosis (≥ grade 1 according to the Desmet classification) in the non-tumorous liver tissue of the resected tissue samples and patients with no signs of chronic liver damage. Interestingly, no significant differences became apparent in these analyses (miR-122: p = 0.758, miR-192: p = 0.802, miR-29b: p = 0.895, miR-155: p = 0.612, U-test) arguing that pre-existing liver diseases had no influence on miRNA serum levels in our cohort of patients. Finally, we evaluated if preoperative portal vein embolization (PVE) had an impact on circulating miRNA levels. However, we observed no significant difference in miRNA serum levels between PVE and non-PVE patients (miR-122: p = 0.893, miR-192: p = 0.976, miR-29b: 0.964, miR-155: p = 0.414, U-test).

For a small number of patients (n = 33–40), miRNA serum concentration after tumor resection were available. We therefore tested if post-operative measurements of circulating miRNAs correlated better with disease characteristics. However, we observed no alterations of serum miR-122, miR-192, miR-29b and miR-155 between different tumor localizations, different T-stages, nodal negative and positive as well as non-metastasized and metastasized disease. Similarly, post-operative miRNA levels were unaltered between R_0_ and R_1_ resected patients (miR-122: p = 0.386, miR-192: p = 0.905, miR-29b: 0.370, miR-155: p = 0.921, U-test).

### Initial miRNA serum levels are unsuitable to predict patients’ prognosis

**Fig 3:**
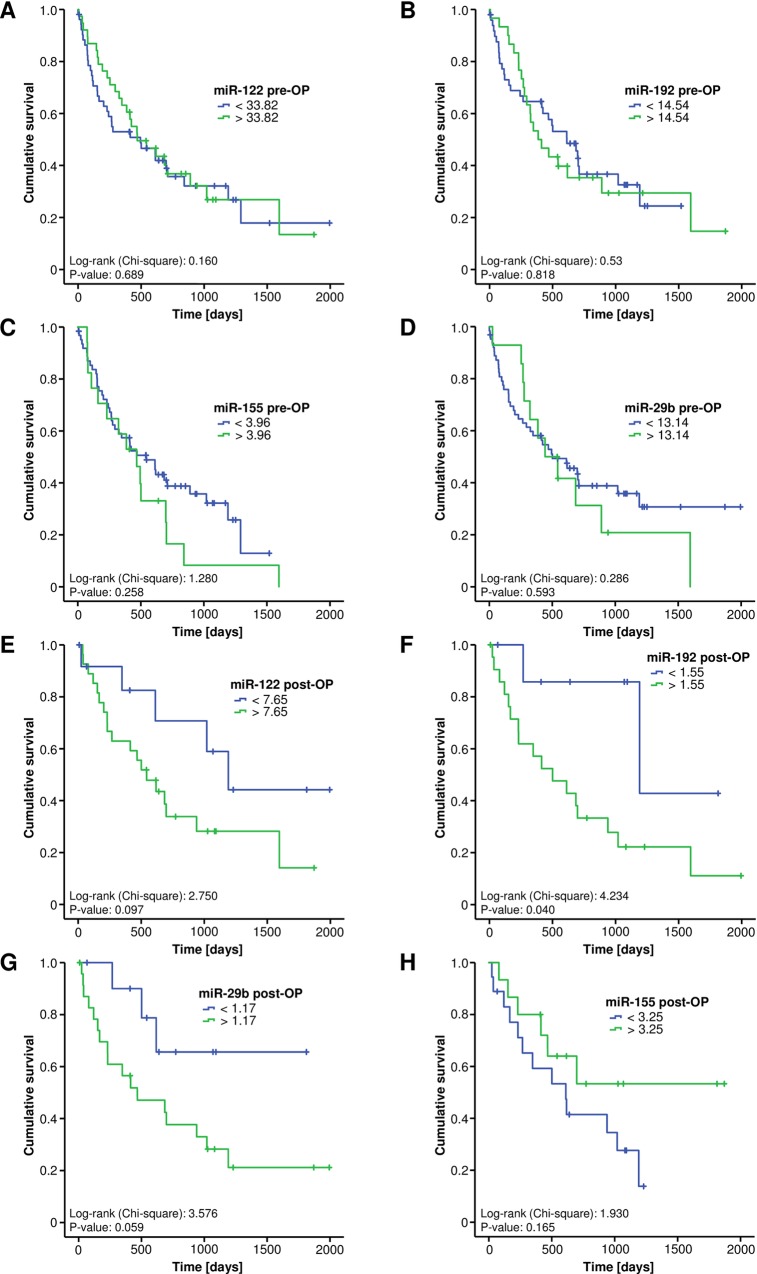
miRNA serum levels and patients’ prognosis. Kaplan-Meier curve analyses show no differences regarding the overall survival between patients with preoperative (pre-OP) serum levels above or below the optimal cut-off value (A-D). High post-operative serum levels of miR-122 and miR-29b show a non-significant trend towards an impaired prognosis (E and G). High serum levels of miR-192 after tumor resection are associated with a significantly impaired long-term survival (F). Post-operative serum levels of miR-155 showed a non-significant trend towards a better outcome in patients with higher serum levels (H).

We subsequently evaluated if serum levels of the four investigated miRNAs might reflect patients’ prognosis after tumor resection. Therefore, we established optimal prognostic cut-off values for each miRNAs to predict an impaired prognosis using the Youden-Index methods. However, Kaplan-Meier curve analyses showed no differences regarding the long-term survival between patients with serum levels above or below the respective cut-off value (Fig 3A–3D). In line, univariate Cox-regression analyses revealed that circulating levels of all four analyzed miRNA before surgery had no impact on the patients’ prognosis after tumor resection (**miR-122:** HR: 0.901 [0.540–1.504], p = 0.690; **miR-192:** HR: 1.069 [0.606–1.886], p = 0.818, **miR-29b:** HR: 1.202 [0.612–2.360], p = 0.593; **miR-155:** HR: 1.421 [0.770–2.622], p = 0.261).

### Elevated levels of serum miR-192 after tumor resection are associated with an unfavorable prognosis

Subsequently, we analyzed if postoperative (post-OP) serum measurement of miRNAs are superior to the initial measurement for the prediction of overall survival. Interestingly, when using the ideal cut-off value which we again determined using the Youden-Index method, we found a significantly impaired long-term survival for patients with high serum levels of miR-192 in Kaplan-Meier curve analysis (Fig 3F) as well as a non-significant trend towards early decease in patients with high levels of miR-122 and miR-29b (Fig 3E and 3G). Postoperative serum levels of miR-155 showed a non-significant trend towards a better outcome in patients with higher serum levels (Fig 3H). Univariate Cox-regression analyses confirmed these results showing a strong trend towards an impaired prognosis in patients with high post-operative levels of miR-192 above the ideal cut-off value (HR: 4.141 [0.954–17.981, p = 0.058]).

### A strong post-operative decrease of miR-122 serum levels is associated with a good prognosis after tumor resection

**Fig 4:**
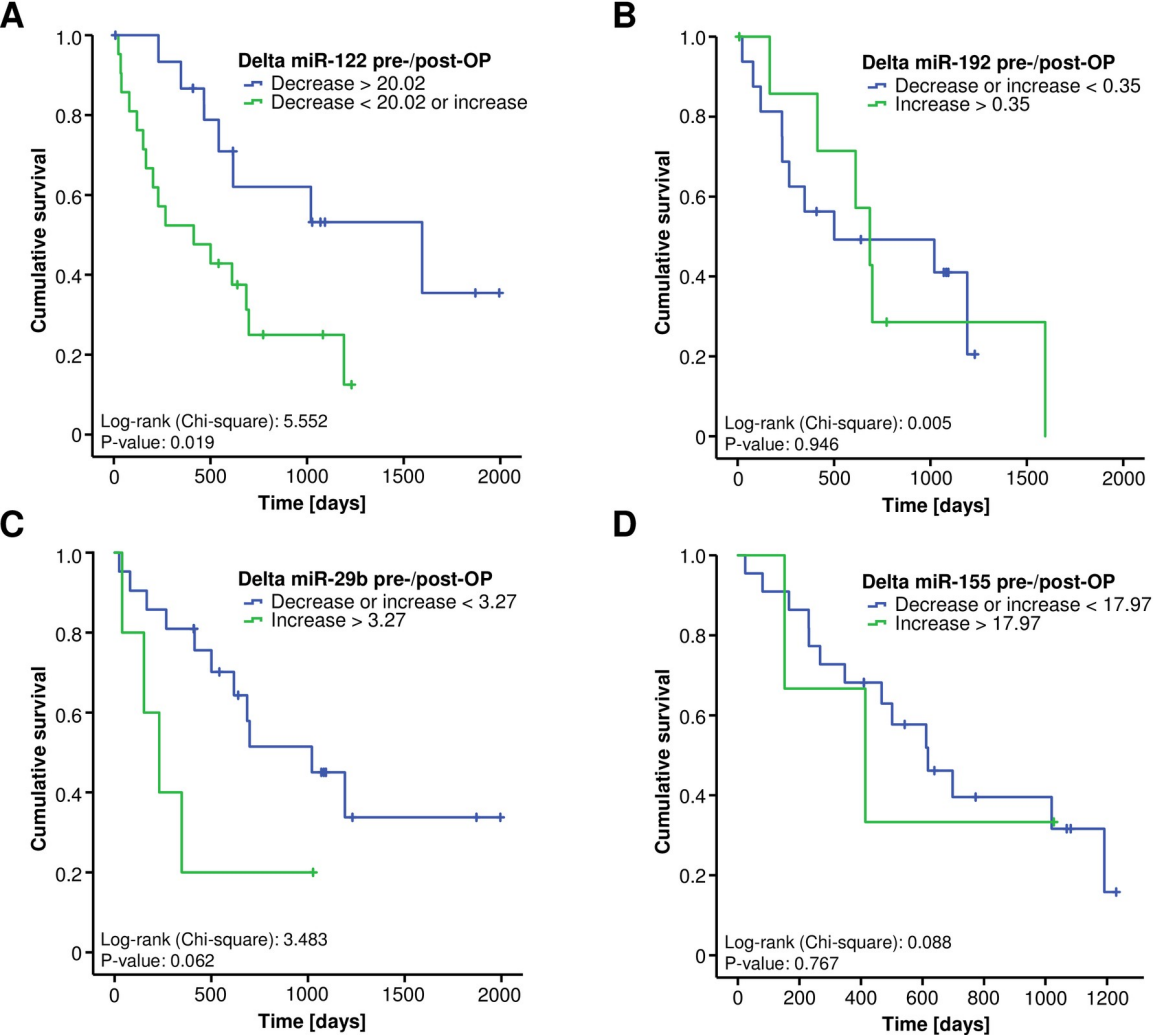
A strong post-operative decrease of miR-122 serum levels is associated with a good prognosis. Kaplan-Meier curve analysis shows that patients with a strong postoperative reduction of miR-122 have a significantly better median overall survival compared to patients with increasing or only slightly decreasing postoperative levels of miR-122 (A). A similar trend of a better prognosis after tumor resection can be found for a strong postoperative decrease of miR-29b (not significant, C), whereas longitudinal changes of serum miR-192 and miR-155 do not reflect patients’ prognosis (B and D).

Finally, we investigated if longitudinal changes of circulating miRNAs before and after surgery can predict patients’ survival. When comparing miRNA serum levels before and after surgery only serum miR-122 levels showed a significant postoperative decrease (S2A Fig) whereas serum levels of miR-192, -29b and -155 were unaltered (S2B–S2D Fig). Interestingly, a strong post-operative reduction of miR-122 was associated with a good prognosis, as Kaplan-Meier curve analysis showed that patients with a postoperative reduction of miR-122 above the ideal cut-off value had a significantly better median overall survival compared to patients with increasing or only slightly decreasing postoperative miR-122 levels (1596 vs. 414 days, Fig 4A). This finding was further confirmed by univariate Cox-regression analysis showing a HR of 2.949 [1.147–7.584] (p = 0.025) for the prediction of long-term survival for patients with a strong decrease of circulating miR-122 after tumor resection. Importantly, uni- and subsequent multivariate Cox-regression analysis including markers of liver and kidney function (AST, bilirubin, creatinine), tumor markers (CEA) as well as markers of systemic inflammation (CRP) revealed that the prognostic impact was independent of these parameters (S2 Table). A similar prognostic trend for a better prognosis after tumor resection was observed for a strong postoperative decrease of miR-29b (not significant, Fig 4C), whereas longitudinal changes of serum miR-192 and miR-155 did not reflect patients’ outcome after tumor resection (Fig 4B and 4D).

## Discussion

In the present study, we demonstrate that pre-operative levels of circulating miR-122, miR-192, miR-29b and miR-155 are elevated in patients with cholangiocarcinoma when compared to healthy controls or PSC patients without malignant transformation. Importantly, a strong postoperative decrease of miR-122, when compared to pre-operative levels, was associated with a favorable patients’ prognosis, indicating that the analysis of circulating miRNAs might not only be helpful when performed for diagnostic purposes but also to predict patients’ outcome after extended liver surgery.

Compared to other gastrointestinal tumors, cholangiocarcinoma represents a rare malignancy [31]. However, its global incidence has risen over the last decades while therapeutic options have remained poor due to advanced stage of disease at diagnosis [3,32]. Thus, only few patients are eligible for curative treatment approaches such as extended liver resections or, in selected cases, liver transplantation [33]. Thus, early detection of CCA is essential to provide patients with an optimal therapeutic approach. At present, CEA and CA19-9 are the most widely used tumor markers in patients with CCA but both markers have only a limited diagnostic power with a rather low specificity [5,6]. In this context, novel classes of biomarkers are urgently needed to facilitate early diagnosis in these patients. MiRNAs, a class of highly conserved noncoding RNAs, have been shown to be involved in the regulation of multiple functions of mammalian cells including cellular development, differentiation, proliferation, and apoptosis [9–11]. Moreover, miRNAs have been described and quantified in serum and blood, where they might serve as biomarkers for various diseases such as cancer [34]. In the context of CCA only very few studies on a potential role of circulating miRNAs are available and delivered highly inconsistent and partially even contradictory results [20,35–38]. Despite methodical and technical factors (differences in standardization, normalization, sample acquisition and storage) this tremendous lack of consistency seems to be a result of relatively small and heterogeneous cohorts. In the present study, we provide data from a large and very well characterized cohort of 94 patients with CCA that underwent extensive liver surgery at a tertiary medical center. We analyzed a biological plausible panel of miRNAs as all of the miRNAs have been shown to be involved in the development or progression of various types of cancers including many gastrointestinal malignancies [39,40–49]. Most importantly, miR-122 and miR-192, which are selectively expressed in hepatocytes, represent the most abundant hepatic miRNAs accounting for up to 70% of all miRNAs of the liver. MiR-122 plays critical roles in liver homeostasis and metabolism. Silencing of miR-122 has been associated with steatohepatitis, fibrosis and carcinogenesis [9, 10]. A liver-specific knockout for miR-122 develops HCC by modulating expression of CyclinG1, ADAM10, IGF1R, SRF and Wnt1 [49]. A down-regulation of miR-29, which is expressed at high levels in primary cholangiocytes [50,51], has been shown to promote carcinogenesis in immortalized, but non-malignant (H69 cholangiocyte) as well as in malignant (KMCH) cholangiocarcinoma cell lines via regulating Mcl-1 expression [52]. MiR-155 was found to be part of a 6 miRNA signature, deregulated in patients with CCA [48]. Thus analysis of these miRNAs, which all reflect specific aspects in the pathophysiology of CCA seemed promising to detect the presence of disease on one hand and to determine patients´ prognosis on the other. Strikingly, we found that serum concentrations of all of these miRNAs were elevated in patients with CCA, with the most prominent increase found for miR-122 (see Fig 1). When analyzed as a single marker, miR-122 demonstrated an extraordinary value to discriminate between CCA and controls (AUC 0.992), which was even superior to that of CA19-9 or CEA, representing the most frequently used marker-proteins for CCA. Importantly, the diagnostic power of circulating miRNAs was even further increased when combining more than one miRNA (see Fig 1F). With respect to potential co-expression pattern of the analyzed miRNAs, we and others have recently demonstrated that serum levels of miR-122 demonstrate a strong correlation to those of miR-192, another liver specific miRNA [53]. Interestingly, also in this study, serum levels of both miRNAs were in strong correlation (see Fig 1H) and miR-192 could also be identified as a diagnostic marker for CCA. Moreover, we observed a strong correlation between miR-29b and mirR-122/192, while miR-155 did not show any correlation. This data is in good agreement with previous studies showing a positive correlation of miR-29 and miR-122 in other hepatic disease such as chronic hepatitis B [54]. Importantly, concentrations of circulating miRNAs are regulated by very different processes of which most are only poorly understood or completely unknown. Our study highlights that not only the tumour burden according to the TNM classification but rather other tumour associated processes represent the main regulators of serum levels of miR-122, miR-192, miR-29b and miR-155 in the context of CCA. Moreover, a distinct miRNA might be regulated in at least partially differential ways by various physiological and pathophysiological influences. It seems therefore likely that the expectable reduction of miR-155 serum levels after tumour resection is masked by postoperative inflammation, representing a different cause of elevated miR-155 serum concentrations.

In clinical routine, early diagnosis of CCA represent an important challenge in PSC patients, which are at high risk for developing a CCA in their chronically inflamed bile ducts. Besides regular imaging, analysis of CA19-9 represents an important element in the screening process of these patients. However, CA19-9 concentrations might be strongly biased by different conditions such as inflammation or cholestasis [5]. We therefore hypothesized that analysis of circulating miRNA might outperform CA19-9 in this setting and performed ROC analysis comparing the AUC values of miR-122 and miR-192 to CA19-9 and CEA. Although statistical significance was not reached, miR-122 showed the highest AUC value in this analysis. In summary, our data suggest a potential of circulating miRNAs in (early)-detecting CCA even in patients with PSC. However, larger clinical trials including a higher number of CCA and especially PSC patients are warranted to fully elucidate the diagnostic relevance of miRNAs in the context of CCA and to particularly investigate previously unrecognized pattern of miRNA co-expression and/or interaction which might yield important information for the diagnostic work-up of this malignancy.

Tough circulating miRNAs could be used as prognostic biomarkers for various types of cancers, in this study we did not detect relevant differences in pre-operative miRNA serum levels between patients that displayed long term survival or those that succumbed to death. Surprisingly, we detected that the difference between pre- and postoperative miR-122 and miR-29b (non-significant trend) serum levels were associated with patients’ survival as those patients with a stronger decrease in post-operative miR-122 survived significantly longer than patients with an increase or only a slight decrease of the respective miRNAs. Furthermore, patients with high levels of miR-192 post-operatively showed a significantly shorter median survival compared to patients with low levels. Such data might be of special relevance for identifying those patients with a need for an additional postoperative treatment such as adjuvant chemotherapy as it seems likely that those patients with a more aggressive disease (which therefore show only a small decrease in postoperative miRNA levels) would particularly benefit from such therapy. However, in order to potentially classify patients into post-operative risk groups according their miRNA levels after surgery or their individual miRNA kinetic before and after surgery, larger clinical trials should be conducted to further unravel potential prognostic capabilities of circulating miRNAs in the context of CCA resection.

There are several limitations in the design of this study. As pointed out, none of the analyzed miRNAs is a specific CCA marker. It is thus possible that an undocumented concomitant disease might have biased their concentrations in the serum. In addition, postoperative serum samples were not available for all patients. Thus, the number of confounders included into the multivariate Cox-regression analysis regarding the prognostic relevance of strongly decreasing miR-122 levels after surgery was limited. Moreover, our study is limited by its retrospective nature and we did only consider the overall survival but not the tumor-free survival, which could be more useful to elaborate the prognostic relevance of miRNA. Finally, our control populations of healthy blood donors and PSC patients showed a significantly younger age compared to CCA patients which might have resulted in an age-related bias on circulating miRNA levels. Thus, larger and prospective trials are needed to nail down the potential of circulating miRNAs in diagnostic and estimation of prognosis in the context of CCA.

## Supporting information

S1 Table Binary logistic regression analysis for the diagnosis of CCA compared to healthy control patients. (DOCX)

S2 Table Uni- and Multivariate Cox-regression analysis for the prediction of overall survival. (DOCX)

S1 Fig Serum levels of miRNAs and disease characteristics. There are no significant differences of serum miR-122 (A), miR-192 (B), miR-29b (C) and miR-155 (D) levels between patients with intrahepatic CCA, Klatskin tumor, distal CCA or gallbladder carcinoma (H-Test). Levels of circulating miR-122, miR-192, miR-29b and miR-155 are unaltered between patients with different T-status (T1-T4, E-H, H-Test), nodal negative and positive (N0 vs. N1, I-L, U-Test) as well as non-metastasized and metastasized disease (M0 vs. M1, M-P, U-Test).
(DOCX)

S2 Fig Serum miRNA levels before and after surgery. Only serum miR-122 (A) levels show a significant postoperative decrease, whereas serum levels of miR-192, -29b and -155 are unaltered before and after surgery (B-D, Wilcoxon signed-rank test).
(DOCX)

## Abbreviations

ALP: Alkaline phosphatase
ALT: Alanine-Aminotransferase
AST: Aspartat-Aminotransferase
BMI: body mass index
CA19-9: carbohydrate-Antigen 19–9
CCA: cholangiocarcinoma
CEA: carcinoembryonic antigen
CRP: C-reactive protein
GGT: Gamma-glutamyltransferase
HR: Hazard Ratio
H-Test: Kruskal-Wallis-Test
miR/miRNA: microRNA
OR: Odds Ratio
PSC: primary sclerosing cholangitis
PVE: portal vein embolization
ROC: receiver operating characteristic
U-Test: Mann-Whitney-U-Test
