# Supplementary material for: Serum levels of miR-29, miR-122, miR-155 and miR-192 are elevated in patients with cholangiocarcinoma
Source: PLoS One. 2019 Jan 17;14(1):e0210944. doi: 10.1371/journal.pone.0210944 (PMC6336320; doi:10.1371/journal.pone.0210944)
Supplement: S1 Table — (DOCX) [file pone.0210944.s001.docx]

## **S1 Table**

| **Parameter** | **Univariate analysis** | | **Multivariate analysis** | |
| --- | --- | --- | --- | --- |
|  | *Odds ratio [95% CI]* | *p=value* | *Odds ratio [95% CI]* | *p=value* |
| **miR-122** | 4.362 [1.964-9.686] | <0.001 | **15.960 [1.106-230.313]** | **0.042** |
| **miR-192** | 2.365 [1.405-3.981] | 0.001 | **4.548 [1.032-20.042]** | **0.045** |
| **AST** | 1.063 [1.026-1.100] | 0.001 | 0.944 [0.888-1.003] | 0.064 |
| **ALP** | 1.065 [1.036-1.095] | <0.001 | 1.028 [0.951-1.111] | 0.491 |

AST: Aspartat-Aminotransferase, ALP: Alkaline phosphatase, miR: microRNA
