# Supplementary material for: Serum levels of miR-29, miR-122, miR-155 and miR-192 are elevated in patients with cholangiocarcinoma
Source: PLoS One. 2019 Jan 17;14(1):e0210944. doi: 10.1371/journal.pone.0210944 (PMC6336320; doi:10.1371/journal.pone.0210944)
Supplement: S2 Table — (DOCX) [file pone.0210944.s002.docx]

## S2 Table

| **Parameter** | **Univariate Cox-regression** | | **Multivariate Cox-regression** | |
| --- | --- | --- | --- | --- |
|  | *HR [95% CI]* | *p=value* | *HR [95% CI]* | *p=value* |
| **∆miR-122 >20.02** | 2.949 [1.147-7.584] | 0.025 | 3.870 [1.268-11.816] | 0.017 |
| **CEA** | 1.005 [1.001-1.010] | 0.021 | 1.006 [0.999-1.013] | 0.092 |
| **CRP** | 1.013 [1.002-1.024] | 0.016 | 1.013 [1.002-1.024] | 0.019 |
| **Creatinine** | 1.087 [0.225-5.251] | 0.917 |  |  |
| **Bilirubin** | 0.989 [0.897-1.090] | 0.828 |  |  |
| **AST** | 1.001 [0.996-1.006] | 0.734 |  |  |

CEA: Carcinoembryonic antigen, CRP: C-reactive protein, AST: Aspartat-Aminotransferase, miR: miRNA
