# Supplementary figures and images for: Serum levels of miR-29, miR-122, miR-155 and miR-192 are elevated in patients with cholangiocarcinoma
Source: PLoS One. 2019 Jan 17;14(1):e0210944. doi: 10.1371/journal.pone.0210944 (PMC6336320; doi:10.1371/journal.pone.0210944)

## S1 Fig

*
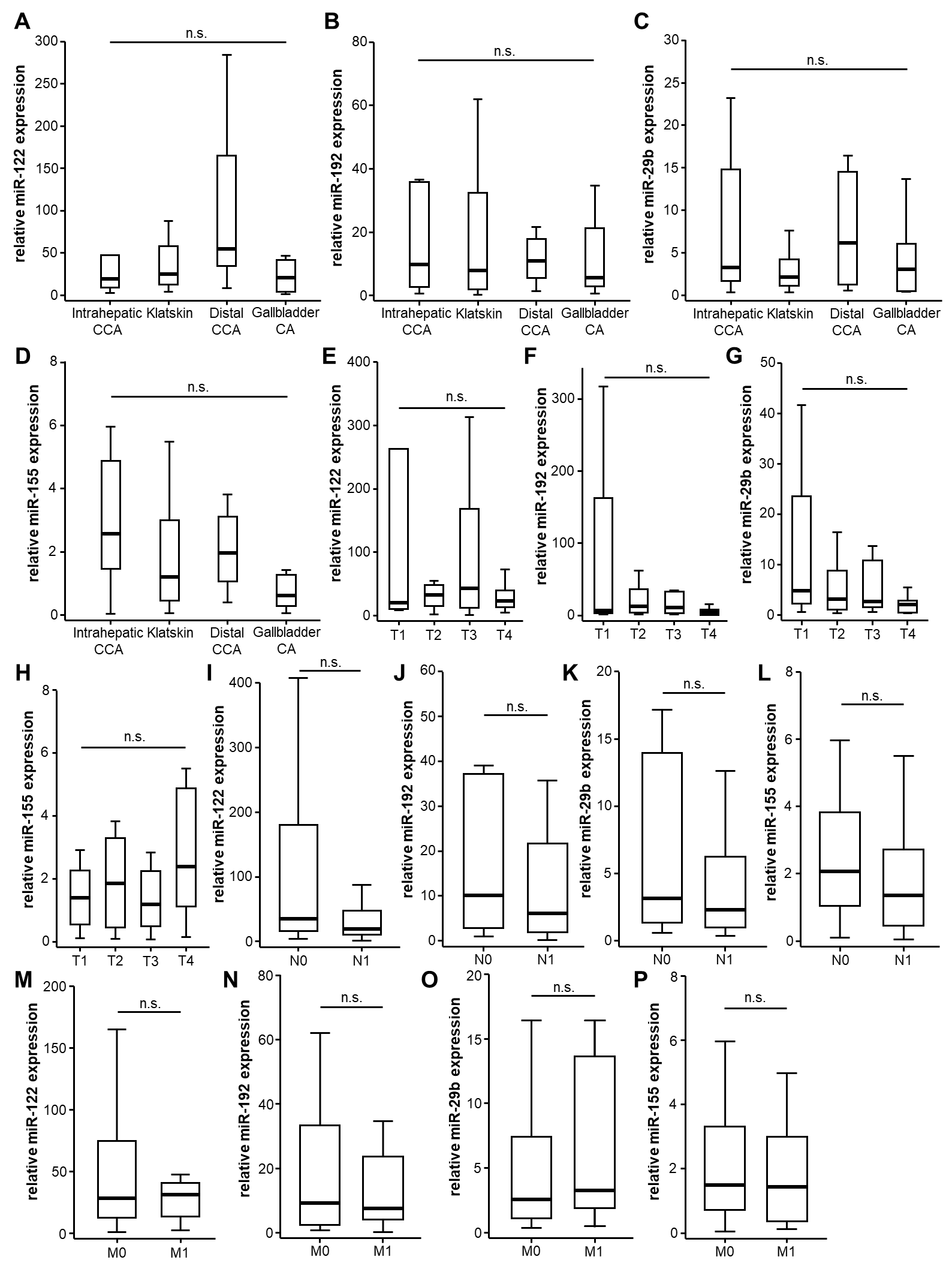
*

Supplement: S1 Fig — There are no significant differences of serum miR-122 (A), miR-192 (B), miR-29b (C) and miR-155 (D) levels between patients with intrahepatic CCA, Klatskin tumor, distal CCA or gallbladder carcinoma (H-Test). Levels of circulating miR-122, miR-192, miR-29b and miR-155 are unaltered between patients with different T-status (T1-T4, E-H, H-Test), nodal negative and positive (N0 vs. N1, I-L, U-Test) as well as non-metastasized and metastasized disease (M0 vs. M1, M-P, U-Test). (DOCX) [file pone.0210944.s003.docx]

## S2 Fig

## *
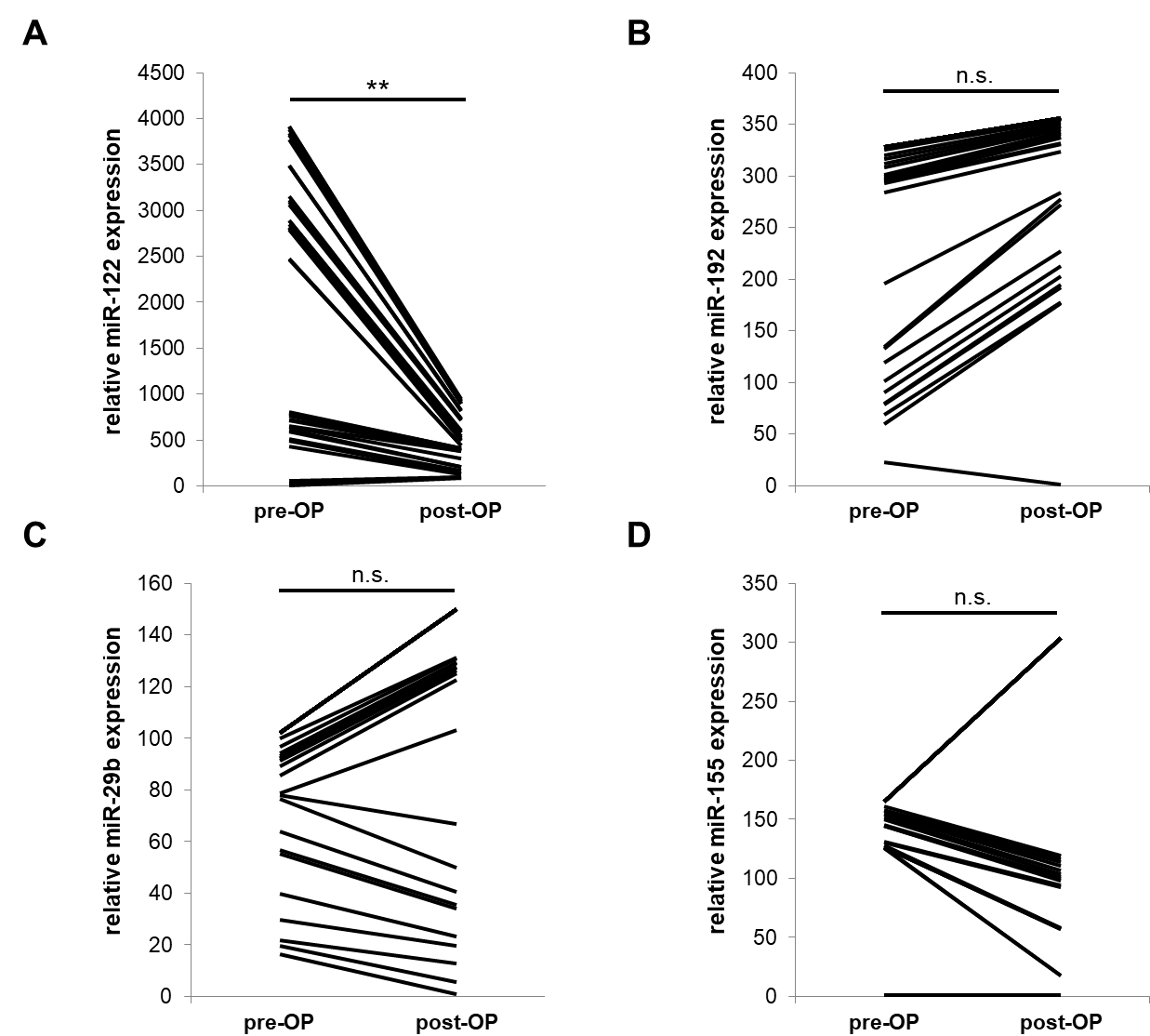
*

Supplement: S2 Fig — Only serum miR-122 (A) levels show a significant postoperative decrease, whereas serum levels of miR-192, -29b and -155 are unaltered before and after surgery (B-D, Wilcoxon signed-rank test). (DOCX) [file pone.0210944.s004.docx]
